# Plasma Diagnostics

**DOI:** 10.3390/s24103257

**Published:** 2024-05-20

**Authors:** Bruno Gonçalves

**Affiliations:** Instituto de Plasmas e Fusão Nuclear, Instituto Superior Técnico, Universidade de Lisboa, 1049-001 Lisbon, Portugal; bruno@ipfn.tecnico.ulisboa.pt

Plasma science and engineering is a multidisciplinary area encompassing some of the most exciting fundamental and applied research themes in today’s scientific landscape, with an extraordinarily broad impact in science, technology, and industry. Although the mainstream areas of plasma research are readily identified as fusion (i.e., magnetic and inertial), laser–plasma interactions, and low-temperature plasma technologies, plasma science is often a key component of many other disciplines, including nanoscience, atomic and molecular physics, surface physics, biophysics, astrophysics, and space science.

The measurement of the parameters of plasmas, usually termed plasma diagnostics, is vital in the quest to harness the power of plasma. Its importance spans across various fields and applications, making it a cornerstone of plasma physics research. Measuring the parameters of plasmas is a key challenge in all these applications, providing essential data for understanding plasma behavior and the basic principles, validating theoretical models, and, in many cases, optimizing and controlling processes in plasma-based applications. In fusion energy research, accurate plasma diagnostics are key to optimizing the conditions for nuclear fusion. In space exploration, understanding plasma properties helps predict and mitigate the effects of space weather on satellites and spacecraft or optimize shielding for spacecraft reentry into the planet’s atmosphere. In industrial applications, plasma diagnostics aid in improving processes like plasma etching and deposition in semiconductor manufacturing.

The range of different methods in plasma diagnostics is necessary to cover this diversity of applications. Each method offers unique insights into the complex behavior of plasmas under different conditions, contributing to the advancement of plasma physics and its applications. By analyzing parameters such as electron density, ion temperature, and electric potential, among many others, researchers can gain valuable insights into the complex phenomena occurring within plasma. Plasma diagnostics is based on a wide variety of characteristic plasma phenomena, and although most of the techniques used are already well established [1], plasma diagnostics is still a very challenging and vivid discipline. On one hand, there is a continuing effort to attain better spatial and temporal resolution, to reach higher accuracies, and to measure with more spatial channels. On the other hand, diagnostic techniques based on more subtle physical processes (compared to those used in routine diagnostics) are continuously being developed, and new tools are being added (e.g., machine learning techniques [2]). Furthermore, to obtain a better insight into the processes taking place in the plasma, it is a prerequisite that plasma parameters are diagnosed simultaneously, as much as possible, with multi-channel diagnostics, preferably with temporal and spatial resolutions smaller than the typical time and length scales of the instabilities.

In some areas, e.g., future fusion reactors [3], such as ITER and future power plant demonstrators, commonly known as DEMO [4,5,6], there will be a need to measure a wide range of plasma parameters in extreme conditions [7,8] of temperature, neutron, and gamma fluxes while providing inputs to control systems [9] with adequate reliability and long-term stability, enabling us to reach and sustain high levels of fusion power in a stationary manner. In such environments, the design of plasma diagnostics becomes a highly interdisciplinary endeavor [10]. The diagnostic design must adapt to the limited space restrictions for the integration of diagnostic components, and strong adverse effects acting on the diagnostic front-end components (neutron and gamma radiation, heat loads, erosion, and deposition) will degrade the diagnostic components over time. Moreover, the nuclear environment of DEMO imposes that any maintenance must be performed by remote handling, making it technically challenging to design diagnostic components for remote handling compatibility. Furthermore, all in-vessel components should be designed for a high degree of durability and reliability to minimize interventions for scheduled and non-scheduled maintenance. All these restrictions are highly demanding on the diagnostic design.

This Special Issue includes sixteen papers focused on the latest advancements in the field of plasma diagnostics, covering methods, instruments, and experimental techniques used to measure the properties of plasma, such as diagnostics for magnetic confinement fusion, beam plasmas and inertial fusion, low-temperature and industrial plasmas, reentry plasmas, and basic and astrophysical plasmas. Each of the sixteen original contributions accepted for publication has undergone a rigorous review process by a minimum of two expert reviewers across at least two rounds of revision and was judged by their technical merit and relevance. These studies published in the current collection are briefly summarized below.

In contribution 1, the authors address the development, design, and commissioning of a diagnostic system for a non-intercepting direct measure of the SPIDER ion source beamlet current, the so-called beamlet current monitor (BCM), aimed at directly measuring the electric current of a particle beam. Stable and uniform beams with low divergence are required in particle accelerators; therefore, beyond the accelerated current, measuring the beam current spatial uniformity and stability over time is necessary to assess the beam performance, since these parameters affect the perveance and thus the beam optics. For high-power beams operating with long pulses, it is convenient to directly measure these current parameters with a non-intercepting system due to the heat management requirement. Such a system needs to be capable of operating in a vacuum in the presence of strong electromagnetic fields and overvoltages due to electrical breakdowns in the accelerator.

In contribution 2, the authors examine the specifications and requirements for high-speed flow measurements for the plasmas produced in the European Shock Tube for High-Enthalpy Research (Portugal). This new state-of-the-art facility is tailored for the reproduction of spacecraft planetary entries in support of future European exploration missions. High-speed events, such as planetary entry shock waves, are very challenging to examine in shock tube facilities owing to their very short timescales (in the order of the μs), hence mandating the deployment of fast diagnostic techniques. The importance of examining the different spectral regions lies in characterizing the physical and chemical processes governing the behavior of the entry plasmas to perfect the numerical models. This paper discusses the design choices for the main diagnostics, with particular focus on VUV-to-IR emission spectroscopy and interferometry diagnostics. The spectroscopy setup covers a spectral window between 120 and 5000 nm, and the microwave interferometer can measure electron densities up to 1.5 × 10^20^ electrons/m^3^.

Contribution 3 addresses the main challenges in the development of a plasma diagnostic and control system for a nuclear fusion DEMOnstration reactor (DEMO). The diagnostics need to cope with unprecedented radiation levels in a tokamak during long operation periods. This paper provides a broad overview of the radiation environment that diagnostics in DEMO are expected to face. Using the water-cooled lithium lead blanket configuration as a reference, neutronics simulations were performed for pre-conceptual designs of in-vessel, ex-vessel, and equatorial port diagnostics representative of each integration approach. Resorting to diagnostics representative of different integration approaches in DEMO—inner vessel, ex-vessel, and equatorial ports—neutronics simulations were performed to estimate the fluxes, heat loads, dose rates, and displacements per atom (dpa) in different sections of the tokamak using pre-conceptual CAD models of the diagnostics. Flux and nuclear load calculations are provided for several sub-systems, along with estimations of radiation streaming to the ex-vessel for alternative design configurations. The results can be used as a reference by diagnostic designers.

In contribution 4, the authors focused on the use of spectroscopy of laser-induced dielectric breakdown plasma in mixtures of air with inert gases Ar, He, Kr, and Xe. The generation of ozone and nitrogen oxides by laser-induced dielectric breakdown (LIDB) in mixtures of air with noble gases Ar, He, Kr, and Xe is investigated using OES and IR spectroscopy, mass spectrometry, and absorption spectrophotometry. It was shown that the addition of He to air does not fundamentally change the spectral pattern of air. In contrast, the addition of Ar suppresses the N+ band at 463.0 nm, while the other bands of nitrogen ions slightly decrease. The addition of Kr leads to even greater suppression of the line intensities of nitrogen ions as well as oxygen ions. It should be noted that when He, Ar, and Kr are added, the atomic oxygen line retains a low intensity. The addition of Xe results in complete suppression of the air component bands. It is expected that this method will be used on real systems, for example, for the treatment of abiogenic media obtained after irradiation with gas mixtures (for example, saline), with a further transition to blood treatment in order to increase its antioxidant potential.

Contribution 5 shows the first results of the implementation of the Doppler backscattering diagnostic (DBS) for the investigation of the transition to H-mode in the spherical tokamak Globus-M2. DBS allows the measurement of the poloidal rotation velocity and the turbulence amplitude of plasma. The multi-frequency DBS system installed on Globus-M2 can simultaneously collect data from different areas spanning from the separatrix to the plasma core, allowing the radial profiles of the rotation velocity and electric field to be calculated before and after the LH transition. DBS measurements of the poloidal plasma velocity and small plasma turbulence allowed for it to be investigated by observing the behavior of these parameters.

Contribution 6 also shows results obtained in Globus-M2. It has been observed that the DBS signal reacts to the backscattering from filaments, which are well known to strongly contribute to particle and energy losses both in L- and H-mode. However, the DBS data have proven difficult to analyze. The contribution focuses on modeling backscattering of filaments using two-dimensional full-wave simulations of backscattering off filaments with the code IPF-FD3D for the interpretation of Doppler backscattering data. This simulation enables us to understand what kind of information can be extrapolated from the signals.

Contribution 7 is focused on refined appearance potential mass spectrometry (APMS) for high-precision radical density quantification in plasma to improve the precision of plasma diagnosis and help elucidate the plasma etching process. As the analysis of complicated reaction chemistry in bulk plasma has become more important, especially in plasma processing, quantifying radical density is now in focus. With a simple modification of the original APMS approach, the fitting process was eliminated, and the He density was obtained over the entire electron energy range. A comparison of the neutral densities in He plasma between the conventional method and the new method, along with the real neutral density obtained using the ideal gas equation, confirmed that the proposed quantification approach can provide more accurate results.

As the importance of ion-assisted surface processing based on low-temperature plasma increases, the monitoring of ion energy impinging on wafer surfaces becomes more important. Monitoring methods that are noninvasive, real-time, and comprise ion collisions in the sheath have received much research attention. Contribution 8 is focused on the development of a noninvasive real-time ion energy distribution (IED) monitoring system based on an ion trajectory simulation where the Monte Carlo collision method and an electrical model are adopted to describe collisions in sheaths. In previous works, IED monitoring systems had the limitations of, for example, neglecting collisions, measuring invasively, and assuming the plasma potential to be a sine wave. To overcome these limitations, the authors investigated the IED measurement with the proposed method and compared it with the results of IEDs measured via a quadrupole mass spectrometer under various conditions. A noninvasive and real-time IED monitoring system was proposed and validated in an asymmetric RF CCP discharge in Ar plasma. The comparison results show that there was no major change in the IEDs as radio-frequency power increased or as the IED gradually became broad as gas pressure increased, which was in good agreement with the results of the mass spectrometer.

Contribution 9 addresses low-temperature plasma diagnostics to investigate the process window shift in plasma etching of SiO_2_. As low-temperature plasma plays an important role in semiconductor manufacturing, plasma diagnostics have been widely employed to understand changes in plasma according to external control parameters, which has led to the achievement of appropriate plasma conditions, normally termed the process window. During plasma etching, shifts in the plasma conditions both within and outside the process window can be observed. In this contribution, the authors utilized various plasma diagnostic tools to investigate the causes of these shifts. Cutoff and emissive probes were used to measure the electron density and plasma potential as indicators of the ion density and energy, respectively, that represent the ion energy flux. Quadrupole mass spectrometry was also used to show real-time changes in plasma chemistry during the etching process. The obtained diagnostic results were able to sufficiently explain the process window shift and, in addition, were in good agreement with the etch model prediction. By extending the SiO_2_ etch model with rigorous diagnostic measurements (or numerous diagnostic methods), more intricate plasma processing conditions can be characterized, which will be beneficial in applications and industries where different input powers and gas flows can make notable differences to the results.

Contribution 10 is focused on the development of a high-linearity voltage and current probe with a floating toroidal coil (FTC). As the conventional voltage and current (VI) probes widely used in plasma diagnostics have separate voltage and current sensors, crosstalk between the sensors leads to degradation of measurement linearity, which is related to practical accuracy. The authors propose a VI probe with a floating toroidal coil that plays both roles as a voltage and current sensor and is thus free from crosstalk. In this paper, the operation principle of the FTC was demonstrated, and its optimum design was established through 3D electromagnetic wave simulation. It is expected that the proposed VI probe could be applicable to plasma diagnostics as well as process monitoring with higher accuracy.

Contribution 11 proposes the measurement of a microwave probe, the measurement of lateral electron density (MOLE) probe, applicable to low-pressure plasma diagnostics. The basic properties of the MOLE probe are analyzed via three-dimensional electromagnetic wave simulation, with simulation results showing that the probe estimates electron density by measuring the surface wave resonance frequency from the reflection microwave frequency spectrum (S11). An experimental demonstration on a chamber wall measuring lateral electron density is conducted by comparing the developed probe with the cutoff probe, a precise electron density measurement tool. Experimental demonstrations, including a cutoff probe for comparison, exhibit that the MOLE probe represents good linearity with the cutoff probe in bulk as well as on the chamber wall, which means that the MOLE probe can measure the lateral electron density. Based on both simulation and experiment results, the MOLE probe is shown to be a useful instrument to monitor lateral electron density.

Contribution 12 is focused on the heavy ion beam diagnostic (HIBD), the only tool for direct measurements of plasma potential in magnetically confined fusion plasmas. The measurements of plasma potential fluctuations are of special interest and importance in investigations of turbulent transport. In a heavy ion beam diagnostic (HIBD), the plasma potential is obtained by measuring the energy of the secondary ions resulting from beam–plasma collisions by an electrostatic energy analyzer with a split-plate detector (SPD), which relates the secondary ion beam energy variation to its position determined by the difference in currents between the split plates. This paper considers the possible influence of the secondary beam non-uniformity on plasma potential and its fluctuation measurements using the SPD technique. The results are supported by experimental data from the tokamak ISTTOK HIBD. 

Contribution 13 goes further in addressing the highlights and recent developments obtained in ISTTOK HIBD. The heavy-ion beam diagnostic installed on the ISTTOK tokamak (Lisbon, Portugal) has been conceptualized to provide simultaneously the plasma radial profile evolution of the plasma temperature, electron density, plasma poloidal magnetic field, and plasma potential. In fact, this diagnostic has the capability to scan the plasma in 2D, although in ISTTOK, it is limited in its coverage by geometrical factors. In practice, it can provide a 1D full-diameter profile of each of the above parameters. This paper describes the capabilities that have been developed in this diagnostic and includes a more detailed description of the physics basis for the measurement of the plasma poloidal magnetic field profile in ISTTOK. ISTTOK HIBD is based on a unique configuration that allows the collection in a multiple-cell array detector of the probing secondary beams generated from the whole plasma diameter. The wealth of information obtained allows for accounting for the path integral effects and retrieving the local values of the plasma parameters at the ionization volume in the plasma. The method allowed for the obtaining of plasma-like pressure profiles with good spectral resolution. Exploitation of the plasma pressure-like measurements in AC discharges allowed for the identification and characterization of MHD activity and turbulent transport in edge polarization experiments. Interesting results are also shown on the real-time determination of the plasma column center, on an innovative method to determine the plasma current poloidal magnetic field, and on the most recent developments in measuring the plasma potential using a cylindrical energy analyzer.

Contribution 14 reports the development, construction, and experimental test of an angle-resolved Thomson parabola (TP) spectrometer for laser-accelerated multi-MeV ion beams that can measure the spectra of discretized beamlets with different emission angles from a laser–plasma interaction experiment at a high repetition rate, simultaneously sorting the ion species by their charge-to-mass ratio. The work presents a multi-pinhole Thomson parabola spectrometer, which combines sharp spectral/angular precision with ionic species sorting capability. A novel analysis method, which can examine the crossing parabolic traces on the detector plane, grants access to several variables simultaneously. The contribution also describes the first test of the spectrometer at the 1PW VEGA 3 laser facility at CLPU, Salamanca (Spain), where up to 15 MeV protons and carbon ions from a 3 μm laser-irradiated Al foil are detected. Such kinds of detectors open interesting prospects for beam analysis of novel acceleration mechanisms, such as collisionless shock acceleration (CSA) or radiation–pressure–acceleration (RPA), as well as for measurements of transported ion beamlines.

Relevant uncertainties in theoretical atomic data are vital to determining the accuracy of plasma diagnostics in several areas, including, in particular, the astrophysical study. Contribution 15 presents a new calculation of the uncertainties on the present theoretical ion-impact charge exchange atomic data and X-ray spectra, based on a set of comparisons with the existing laboratory data obtained in historical merged-beam, cold-target recoil-ion momentum spectroscopy, and electron beam ion traps experiments.

To conclude, contribution 16 addresses the advances, challenges, and future perspectives of microwave reflectometry for plasma position and shape control on future nuclear fusion devices. Microwave reflectometry is a radar-based technique that can be used to determine the radial distribution of plasma density in fusion experiments. This technique has been proposed as an alternative to magnetic measurements for plasma position control. This contribution presents the multiple engineering and physics challenges addressed while designing reflectometry diagnostics using radio science techniques. Specifically, short-range dedicated radars for plasma position and shape control in future fusion experiments, the advances enabled by the designs for ITER and DEMO, and future perspectives. One key development is also in electronics, aiming at an advanced, compact, coherent, fast-frequency sweeping RF back-end (23–100 GHz in a few μs) that is being developed at IPFN-IST using commercial monolithic microwave integrated circuits (MMIC). 

The sixteen contributions in this Special Issue offer invaluable updates and insights into various aspects of plasma diagnostics development, addressing the challenges in these research domains. These findings can serve as a catalyst for future endeavors aimed at enhancing and optimizing next-generation plasma diagnostics. Each contribution reflects the collective ingenuity and relentless efforts of researchers committed to pushing the boundaries of knowledge, underscoring the multidisciplinary nature of the field. 

To conclude, I would like to add a word of heartfelt gratitude to all the authors for their invaluable contributions to this Special Issue, as well as to the reviewers who generously devoted their time and expertise to ensure the quality of the published papers, thus contributing to the success of this endeavor.
